# Industrially Relevant Enzyme Cascades for Drug Synthesis and Their Ecological Assessment

**DOI:** 10.3390/ijms23073605

**Published:** 2022-03-25

**Authors:** Regine Siedentop, Katrin Rosenthal

**Affiliations:** Chair for Bioprocess Engineering, Department of Biochemical and Chemical Engineering, TU Dortmund University, D-44227 Dortmund, Germany; regine.siedentop@tu-dortmund.de

**Keywords:** biocatalysis, in vitro biotransformations, enzyme cascades, multi-enzymatic reactions, active pharmaceutical ingredients, industrial applications, *E*-factor, sustainability

## Abstract

Environmentally friendly and sustainable processes for the production of active pharmaceutical ingredients (APIs) gain increasing attention. Biocatalytic synthesis routes with enzyme cascades support many stated green production principles, for example, the reduced need for solvents or the biodegradability of enzymes. Multi-enzyme reactions have even more advantages such as the shift of the equilibrium towards the product side, no intermediate isolation, and the synthesis of complex molecules in one reaction pot. Despite the intriguing benefits, only a few enzyme cascades have been applied in the pharmaceutical industry so far. However, several new enzyme cascades are currently being developed in research that could be of great importance to the pharmaceutical industry. Here, we present multi-enzymatic reactions for API synthesis that are close to an industrial application. Their performances are comparable or exceed their chemical counterparts. A few enzyme cascades that are still in development are also introduced in this review. Economic and ecological considerations are made for some example cascades to assess their environmental friendliness and applicability.

## 1. Introduction

Industrial production of active pharmaceutical ingredients (APIs) moves towards more sustainable and environmentally friendly processes. Currently, several tens to hundreds of kilogram waste per kilogram product are produced during API synthesis, much of it from organic solvents [1,2]. However, environmental awareness but also regulations by governments increase the demand for greener processes, a trend which started already in the 1990s [3,4]. Many of the aforementioned principles of green chemistry can be addressed by biocatalysis. For example, enzymes work under mild conditions and often, less solvents have to be used than in chemical approaches. In addition, they are non-toxic, non-hazardous, are generated from renewable resources, and are biodegradable, reducing the environmental contamination [5]. Furthermore, considering that most APIs are chiral compounds [6], enzymes bring the advantage of regio-, stereo-, and enantioselectivity. Biocatalysis can be based on either cell-free or whole-cell systems. Both approaches bring their advantages and disadvantages, such as the inexpensive production of required enzymes and cofactors in cell-based biocatalysis or the adjustment of enzyme and cofactor concentrations in in vitro-systems [7]. In this context, metabolic engineering enables the development of customized pathways that open up new possibilities, including the synthesis of APIs [8]. In this regard, cell-free multistep biocatalysis, allows more precise control of system components with fewer side reactions compared to whole-cell-based approaches [9]. The catalysis of several reactions with isolated enzymes in a one-pot fashion is called enzyme cascade or multi-enzymatic reaction. The advantages of such a system are (i) their ability to produce complex molecules from simple building blocks, where a classical synthesis would be more difficult or fail, (ii) a reduced need for intermediate isolation leading otherwise to large amounts of waste and a loss of material, (iii) high conversions and yields with few by-products, (iv) pushing the reaction equilibria towards product side, and (v) an easier product purification [10]. Nonetheless, enzyme cascades play a minor role in the industrial production of APIs until now. Current challenges include productivities and yields achieved with enzyme cascades, and therefore research needs to be focused on optimal enzyme interaction and system optimization [11]. The ability to engineer enzymes to accept non-natural products, achieve higher rates and tolerate different reaction conditions such as solvents or pH values broadens the range of possibilities and increases the chance for industrial applications. Nevertheless, a few multi-enzyme cascades have made it into industrial applications for drug synthesis.

In this review, examples of industrially relevant enzyme cascades for API syntheses are presented and the application and relevance of the compounds are shown. In addition, enzyme cascades for drug synthesis that are emerging and where more research must be conducted are introduced. The APIs of interest show promising pharmaceutical properties and are investigated in clinical trials or are already approved drugs. Ecological metrics are presented for some cascades and economic considerations are included.

## 2. Industrially Relevant Enzyme Cascades for API Synthesis

**Figure 1:**
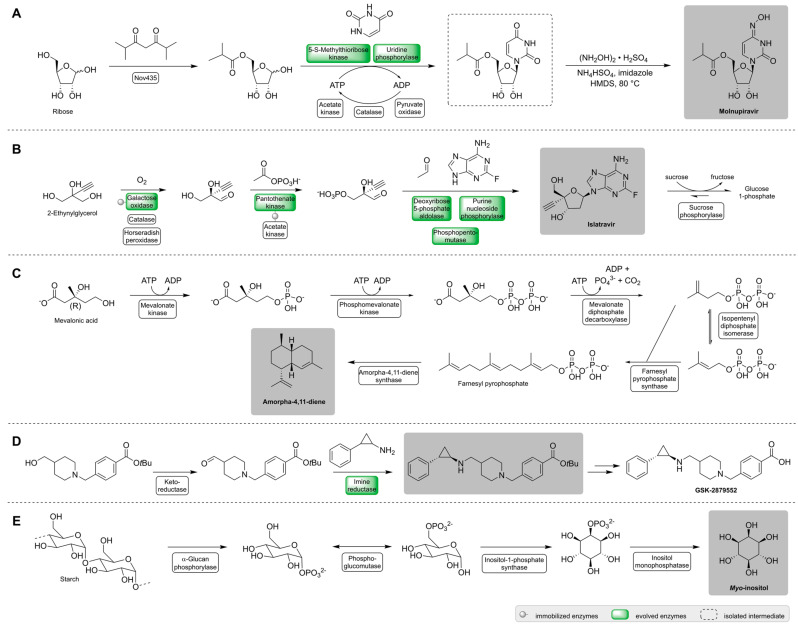
Enzyme cascades for the synthesis of molnupiravir (**A**) [12], islatravir (**B**) [14], artemisinin-precursor amorpha-4,11-diene (**C**) [16], protected GSK-2879552 (**D**) [19], and *myo*-inositol (**E**) [20].

In this section, enzyme cascades are presented, that were developed by companies and covered by patents and are thus close to application. Table 1 and Figure 1 give an overview of the APIs, their production by enzyme cascades and the potential market.

**Table 1:** Overview of APIs with enzyme cascades, their performance, current production, and their potential market.

API	Application	Enzyme Cascade	Performance	Current Production	Market	Ref.
Molnupiravir	COVID-19 treatment	6 enzymes, 2 of them engineered, one isolation step	69% yield	Chemical, 10-step synthesis	409 million confirmed cases of COVID-19 ^1^	[12,13]
Islatravir	HIV treatment	9 enzymes, 5 of them engineered, 3 immobilized	51% yield	Chemical synthesis	37.7 million people were living with HIV in 2020 ^2^; HIV drug market 2020: USD 28.79 billion	[14,15]
Artemisinin	Anti-malarial drug	7 enzymes	~100% conversion, production rate: ~5.7 µmol L^−1^ min^−1^	Plant extraction, semisynthetic production	Malaria cases 2020: 241.000; ACTs sold: >3.5 billion ^2^	[16]
Protected GSK2879552	SCLC and AML treatment	2 enzymes, 1 engineered	5 g scale with 48.3% yield, 99.5% e.e. and 97.9% purity	Chemical synthesis	SCLC: 264,813-331,016 in 2020 ^3^; AML: 21,450 estimated for 2019 [17]; Global oncology drug market 2019: USD 141.33 billion [18]	[19]
*Myo*-Inositol	Application in pharmaceutical, food and feed, and cosmetic industry	4 enzymes	Product concentration: 95 g L^−1^ after 48 h of 125 g L^−1^ maltodextrin (DE ~10), 20,000 L	Chemical dephosphorylation of phytate	15 thousand tons per year, around USD 60 million by 2020	[20,21]

^1^ WHO, 13 February 2022; ^2^ WHO; ^3^ calculated from ^2^; COVID-19: coronavirus disease 2019; HIV: human immunodeficiency virus; ACT: artemisinin-based combination therapy; SCLC: small cell lung cancer; AML: acute myeloid leukemia.

An API that has recently become more important was Molnupiravir (MK-4482, EIDD-2801). Molnupiravir was discovered and developed at Emory Institute of Drug Development (EIDD) in 2013 as anti-viral agent against influenza and gained increased attention in 2020 for the treatment of COVID-19 (coronavirus disease 2019) [22]. COVID-19 is a disease affecting the respiratory system caused by severe acute respiratory syndrome coronavirus-2 (SARS-CoV-2). The virus led to a worldwide and still ongoing pandemic with an increase in mortality and morbidity. Molnupiravir is an orally available prodrug that acts on the RNA-dependent RNA-Polymerase (RdRp) which replicates the viral RNA genome of the coronavirus. Clinical trials of phases 1 to 3 addressed the drug as safe and tolerable in humans and also showed effectiveness in mild, but not moderate-to-severe, COVID-19 cases by reducing the risk of hospitalization [23]. Molnupiravir seems to even be active against newer variants of the coronavirus such as Omicron since it targets conserved genes [24]. Many synthetic routes were already developed for the anti-viral agent, many being chemical approaches [25]. In turn, Merck & Co developed a biocatalytic route, which is shorter and higher yielding than the original, patented chemical synthesis [12]. This current chemical route synthesizes molnupiravir with <10% yield in five steps from uridine, which in turn is synthesized in five steps from ribose and uracil [13]. The biocatalytic route, on the other hand, involves three steps and leads to molnupiravir with an overall yield of 69% (Figure 1A) [12]. Since there is a high demand for this drug, the cascade was developed to use commodity raw materials such as ribose in a sustainable and efficient way. The first two synthesis steps are catalyzed by three enzymes. Two of them were engineered for higher activity by directed evolution with an 80- and 100-fold improvement compared to the original enzymes. Furthermore, a regeneration cascade was implemented for a sufficient ATP supply and phosphate recycling by three additional enzymes. The resulting intermediate is isolated with 87% yield and is further transformed to the final product in the last step. Molnupiravir is received with high yields of 95% for every step and only a low amount of side-products are reported [12]. It is to be mentioned, that the whole cascade was developed in six months showing the impressive potential in enzyme engineering and the development of productions by multi-enzymatic reactions in a rapid manner. This example demonstrates how enzyme cascades can be used to rapidly establish synthetic routes for highly demanded compounds and respond to current needs.

A further impressive example is the synthesis of islatravir (EFdA, MK-8591). Islatravir is an investigational drug for the prevention and treatment of human immunodeficiency virus (HIV) infection [26]. It prevents HIV from multiplying and reduces the amount of the virus in the body by acting as a nucleoside reverse transcriptase translocation inhibitor (NRTTI). In clinical trials, the drug was administered in a daily dose and showed promising effects. However, the study was paused in late 2021 due to a reduction in lymphocyte and CD4 cells in participants, which are important cells for an effective immune response [27]. Nevertheless, the antiretroviral agent will be investigated further [27]. Several chemical syntheses were reported for the compound ranging from 12 to 18 steps [28,29,30,31,32]. In the synthesis of islatravir, the C-4′-stereocenter is a challenging part along with the need of protecting groups [14]. In 2019, a three-step biocatalytic cascade was published by Merck & Co with which the stereocenter was introduced by an enzymatic desymmetrization and no protecting groups and isolation steps were necessary (Figure 1B) [14]. For the design of the synthetic route, a retrosynthetic approach was applied using the bacterial nucleoside salvage pathway as inspiration. This gave ethynyl glycerol as a simple starting material for the synthesis of the deoxyribonucleoside. The overall cascade consists of nine enzymes in one pot of which five enzymes were engineered by directed evolution to act on non-natural substrates and to improve reaction rates as well as the operational stability. For example, for the first enzyme of the cascade galactose oxidase (GOase), 34 amino acids were exchanged in 12 evolutionary rounds resulting in an 11-fold increase in activity. Islatravir was received with 51% yield with high atom economy and efficiency in only three reaction steps. In a further study, the first enzyme was replaced by an asymmetric ketone alkynylation to introduce the stereocenter [33]. This synthesis consisted of eight steps and islatravir could be received in a 120 g scale with a 30% overall yield.

Another important API is the antimalarial drug artemisinin. Artemisinin was discovered in 1972 by Tu Youyou in the plant *Artemisia annua*, for which she was awarded the Nobel Prize in Physiology and Medicine in 2015 [34]. Today, artemisinin-based combination therapies (ACTs) containing artemisinin derivatives and other compounds are the most widely used drug against the parasite *Plasmodium* spp. [35]. The mechanism of action, however, is not yet fully understood. It is concluded that free radicals are formed when the ozonide (trioxane) moiety of artemisinin comes into contact with Fe-ions, which are found in higher concentrations in erythrocytes and plasmodia, which accumulate iron. The free radicals damage susceptible proteins leading to the death of the parasite [36]. The botanical supply of artemisinin with sweet wormwood, natively found in Asia, is inconsistent (prices fluctuated in 2011–2015 between 896.7 and 221.6 USD kg^−1^, respectively [37]) and therefore making other synthetic accesses attractive. The complex structure of artemisinin makes a chemical synthesis difficult [38] and gives bio-based synthetic routes more attention to stabilize the supply. A synthesis of the artemisinin precursor artemisinic acid in yeast by an engineered mevalonate pathway and integrated enzymes to convert amorpha-4,11-diene (AD) to the product was published in 2006 with titers up to 100 mg L^−1^ [39]. The semisynthetic route was improved by J.D. Keasling and Sanofi to produce 50 to 60 tons per year [40,41]. This corresponds to a third of the global annual need of the drug [40,41,42] and production costs are about 400 USD kg^−1^ [43]. However, the semisynthetic artemisinin was still more expensive than agricultural artemisinin leading to the sale of the production plant to Huvepharma [42,43]. Artemisinin production in other plants such as *Nicotiana benthamiana* and *Physcomitrella patens* has also been successful [44]. Nevertheless, other synthetic routes were developed such as an in vitro approach with isolated enzymes. This gives the opportunity for an easier manipulation and optimization of the system than in a cellular environment. In 2013, Chen et al. published an in vitro synthetic route for the production of the artemisinin precursor amorpha-4,11-diene (AD) on a lab scale [45]. The substrate mevalonic acid is converted to farnesyl pyrophosphate (FPP) following the mevalonate pathway using five enzymes. A sixth enzyme, amorpha-4,11-diene synthase (ADS), converts FPP to AD. Enzyme ratios of the cascade were optimized by Taguchi orthogonal array design and response surface methodology, with which inhibitory effects of some enzymes were also identified. This was followed by an additional optimization of the buffer system to increase AD yield. In the end, a conversion of 100%, an almost theoretical yield with 340 mg L^−1^ and an AD production rate of ~2.0 μmol L^−1^ min^−1^ was reached [16]. Later, metabolic engineering revealed further inhibiting effects on the enzymes and bypassing it resulted in a 3-fold enhancement on the AD production rate to ~5.7 µmol L^−1^ min^−1^ [45]. Notably, the study revealed that the enzymes can be reused for up to seven cycles when co-immobilized, providing a good starting point for a large-scale production as it offers many advantages with regard to biocatalyst reuse and the possibility of continuous processes.

An API against cancer is the lysine-specific histone demethylase-1A (LSD1 or lysine demethylase 1A, KDM1A) inhibitor GSK-2879552 [19]. The protein LSD1 has an important role in cancer, infections, or immune modulations, for example. It gained interest as a target in cancer treatment since the inhibition of it leads to impaired cell proliferation, differentiation, invasion, and migration [46,47]. The inhibitor GSK-2879552 was discovered by the screening of a compound library. It was investigated for its activity against small cell lung cancer (SCLC) and acute myeloid leukemia (AML) [46,48]. However, clinical trials for the treatment of SCLC in phase I were terminated in 2019 due to the risk in relapsed refractory SCLC [47]. Nevertheless, other therapeutic applications are still under investigation [49]. In 2019, an enzymatic route was published by GlaxoSmithKline (GSK) for the synthesis of GSK-2879552. This route enables a greener synthetic access with fewer steps, less solvents, reduced costs, and lower process mass intensity (PMI) than the previous chemical approach [19]. The cascade to produce the protected intermediate of GSK-2879552 consists of two enzymes, keto-reductase (KRED) and imine reductase (IRED), which recycle each other’s cofactor NADP(H). To meet several criteria in process conditions, the IRED was evolved by directed evolution in three rounds resulting in a much more active enzyme and meeting or exceeding all their criteria, namely, tolerating a slightly acidic pH, increased substrate concentration and product yield, as well as reduced enzyme loading. The IRED on its own was used for a kilogram scale reaction and the cascade was performed on a 5 g scale with 48.3% yield, 99.5% e.e. and 97.9% purity [19]. This is an exemplary demonstration of large-scale biocatalysis with the economics required for commercial use while providing improved green metrics compared to the chemical synthesis route. A compound with a broad application spectrum is *myo*-inositol. It is used in the food and feed, pharmaceutics, and cosmetic industry. It was once seen as vitamin B_8_, but since it can be produced by the human body, it is not an essential nutrient and therefore no vitamin. Nevertheless, inositol plays an important role in various biological functions and a reduced synthesis ability can result in various diseases. Other species such as birds, fish, shrimps, and some mammals lack the synthetic ability completely [20]. Nutritional supplements are beneficial in these cases. Inositol is also used in the pharma industry for the treatment of, for example, polycystic ovary syndrome (PCOS) [50], and in the food industry as an additive, for example, as a nutraceutical and sweetener. Furthermore, inositol also finds application for the synthesis of inositol nitrate, found in explosives and solid rocket propellants, or glucaric acid, a biopolymer precursor [20]. This results in a world market of 15,000 tons per year and a global market of USD 60 million by 2020 [21]. It is mainly produced from phytate (inositol hexakisphosphate, IP6), the main phosphorous storage of plants. After its extraction, phytate is chemically dephosphorylated, which suffers from various drawbacks such as phosphorous waste, high production costs and difficult *myo*-inositol separation [21]. An alternative route is a cell-based production by metabolically engineered microorganisms such as *Escherichia coli* with a titer of 106.3 g L^−1^ (590.5 mM) and a yield of 0.82 mol mol^−1^ glucose in 23 h [51]. Notably, several in vitro synthetic routes were developed with sugar as starting materials such as starch [20,52], cellulose [53], xylose [54], sucrose [55] or glucose [56]. The first in vitro *myo*-inositol production on an industrial scale was performed by You et al. in 2017 with starch as starting material [20]. On a lab scale, the inositol yield reached 98.9% after optimization of the pathway. The established six-enzyme cascade was used in a 20,000-L bioreactor at 70 °C. A product concentration of 95 g L^−1^ was reached after 48 h with 125 g L^−1^ maltodextrin (DE ~10) as substrate. Since thermophilic enzymes were used, they could be easily purified by heat treatment, which reduced the production costs. This way, the production costs were reduced to about one third of the phytate-based method, demonstrating the industrial usability of enzyme cascades even more [20].

These examples demonstrate the applicability of enzyme cascades for API synthesis in industrial production. The enzymatic synthesis can have higher yields than their chemical counterparts even on a large scale. Reaction steps and the need for intermediate isolation are often reduced resulting in less waste, along with the reduced amount of solvents and often milder reaction conditions such as lower temperatures. With these preceding examples, the way for further enzyme cascades for API synthesis is paved, which might find their eligibility in industry in the future.

## 3. Emerging Enzyme Cascades for API Synthesis

**Figure 2:**
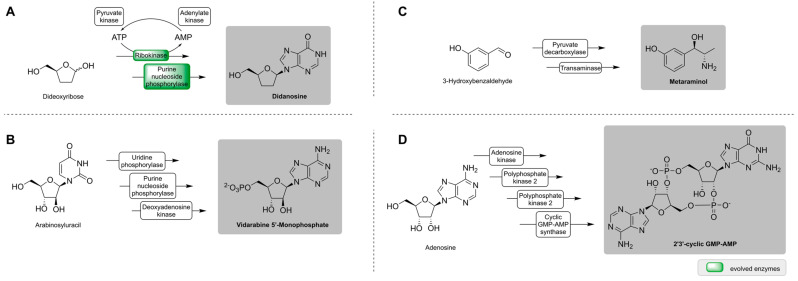
Shortened reactions of the enzyme cascades to produce didanosine (**A**) [57], vidarabine 5′-monophosphate (**B**) [58], metaraminol (**C**) [59], and 2′3′-cyclic GMP-AMP (**D**) [60].

Enzyme cascades for API synthesis are presented that are a step further away from their industrial application. This may be due to their performance, which does not yet meet the criteria of an economic large-scale production. Research is still needed to optimize these systems to achieve higher yielding enzyme cascades. Figure 2 gives an overview of the reactions that are introduced, although there are many more, which are not covered here.

Didanosine (ddI), sold under the name Videx^®^, was the second drug approved for the treatment of HIV in 1991 [61]. It is, however, associated with adverse side effects, especially with long-term exposure [62]. In 2014, a multi-enzymatic route was published for didanosine synthesis by Birmingham et al. (Figure 2A) [57]. Here, a pathway was created using a bioretrosynthetic approach, which was inspired by the sugar and nucleoside metabolism. Initially, the cascade consisted of three enzymes, which were engineered to convert 2′,3′-dideoxyadenine to didanosine for an increased turnover and selectivity for their non-natural substrates. Due to an inhibitory effect of ATP on one enzyme, a regeneration system of the cofactor with two enzymes was included. By engineering one of the pathway enzymes, a shortcut was established. The final cascade of four enzymes produced didanosine with a product titer of 44 µM and 4.4% yield. Even though these are rather low values, this corresponds to a 50-fold increase compared to the original enzymatic route, namely the progenitor pathway. Enzyme engineering is a complex optimization problem, especially when several parameters need to be improved simultaneously to achieve efficient performance of an enzyme cascade. Nevertheless, the example of didanosine synthesis shows the viability, which will certainly lead to further improvements in the future. Another antiviral drug is vidarabine (arabinosyladenine (araA), Vira-A^®^). The API is used to treat the herpes virus and was the first approved nucleoside analogue to be administered systemically [63]. In 2015, a multi-enzymatic synthesis of araA was developed starting from arabinosyluracil (araU) with two enzymes [64]. The enzymes were immobilized, and with optimized reaction conditions, a yield of 53% (3.5 g L^−1^) and 98.7% purity was reached on a 2 L scale. The two enzymes were stable for up to five reaction cycles in the presence of the solvent *N*,*N*-dimethylformamide (DMF). The solvent was necessary since araA has a low solubility in water. To overcome this drawback, the cascade was expanded by the phosphorylation of araA to vidarabine 5′-monophosphate (araA-MP) (Figure 2B) [58]. The third enzyme was also immobilized, and reaction conditions were optimized. On a 10 mL scale with 25 mM AraU, they reached 95.5% conversion after 81 h, corresponding to 82.9 mg of araA-MP [58].

Another enzymatic synthesis of an API that uses a solvent is from Mack et al. from 2021 (Figure 2C) [59]. With a two-enzyme cascade, metaraminol can be produced out of 3-hydroxybenzaldehyde (3-OH-BA). Metaraminol is used to treat hypotension during anesthesia [65]. However, the enzyme cascade contains a transaminase, which often has unfavorable reaction equilibria resulting in low product titers. A possible solution to pull the reaction equilibrium to the product side is in situ product removal (ISPR). ISPR was therefore integrated by liquid-liquid extraction. A screening of solvents for the highest conversion and enzyme activity revealed 1-decanol to be most suitable. With this ISPR concept and an optimized pH value, a yield of 29% with a selectivity of 68% could be reached compared to 14% conversion without ISPR [59].

Becker et al. recently developed an enzyme cascade for the production of 2′3′-cyclic guanosine 5′-monophosphate-adenosine 5′-monophosphate (2′3′-cGAMP) (Figure 2D) [60]. 2′3′-cGAMP is a second messenger that triggers an innate immune response through the release of interferons. It could be used as vaccine adjuvant and derivatives of this natural compound are investigated for their treatment of cancer of which one is already in clinical phase 2 [66,67]. The enzymatic synthesis uses adenosine and guanine 5′-triphosphate (GTP) as starting material. Adenosine is phosphorylated by an adenosine kinase and two polyphosphate kinases (PPKs) to ATP. This way, cheap substrates could be used to gain the high value product. Enzyme and substrate concentrations were rationally and iteratively optimized, with small changes affecting 2′3′-cGAMP production. Using the four-enzyme cascade, 2′3′-cGAMP was produced with 0.08 mole per mole adenosine. Even though this seems low, the yields were comparable to the chemical synthesis starting from phosphoramidite [60].

Although these enzyme cascades are not yet relevant for an industrial application, they show great potential for API syntheses. In addition, they demonstrate the versatility and optimization potential even when using unconventional approaches such as the usage of solvents for ISPR. Although the performance of enzymes is certainly affected by the presence of solvents, from a reaction engineering perspective, the use of solvents is also a good solution to address challenges of substrate and product solubilities. Further research in this area is certainly needed. Nevertheless, as more research is completed in this area, the more opportunities will emerge to customize these systems to one’s need, which will pave the way for industry even further.

## 4. Economic and Environmental Considerations of Enzyme Cascades

In this section, we address the fact that the use of enzyme cascades in drug synthesis is not only beneficial for the accessibility of new molecules, but at the same time can be economically efficient and environmentally friendly. The application of multi-enzymatic reactions in industry inevitably requires strategies to be economically competitive. At the same time, more and more emphasis is being placed on developing sustainable processes in the development of new syntheses. The factors of economics and ecology are proportionally interrelated, especially in the early stages of development, when it is necessary to achieve high overall performance, which includes reaction rates, stabilities, specificities, yields of the overall cascade. The more enzymes involved in a cascade, the more important it becomes that the individual enzymes perform ideally under process conditions.

A major advantage of multi-step reactions in one pot is certainly the reduction in isolation and purification steps, which leads to a significant reduction in waste generation [68]. The combination of several reaction steps in one vessel usually means fewer unit operations, less solvents, and less waste [69]. Simple sustainability considerations, such as the calculation of the environmental factor (*E*-factor, corresponds to kg waste per kg product), show that multi-enzyme cascades can perform appropriately. The development of a biocatalytic process for the preparation of 4-methoxyphenyl-1,2-propanediol, a potential anti-inflammatory drug and valuable synthon for (*R*)-tamsulosin and silibinin, showed *E*-factors of 14 to 46 for different product isomers, which lie in the range of defined benchmarks for drugs (25 to >100, [70]) (Table 2) [71]. These excellent values were achieved with the two-step cascade by assembling the cascade in such a way that coproducts were recycled and could thus be reintroduced. In addition, enzymes were selected that gave products with >99% enantiomeric excess. Previous studies showed similar *E*-factors for the two-step synthesis of vicinal chiral diols [72]. However, when the purification step, in this case chromatography, was included, the *E*-factor increased 90-fold to 1927 kg_waste_ kg_product_^−1^. Therefore, as for other processes, an integrated view of up- and downstream is useful during the development of enzyme cascades for drug synthesis. Few studies go into direct comparison with chemical synthesis. In the case of the synthesis of araA, however, this comparison shows that the use of multi-enzymatic reactions can reduce the *E-*factor to less than one third, indicating the environmental friendliness of the synthesis route [64].

In these very simple calculations, enzyme production is often neglected. Depending on the amount of enzymes used, the contribution of enzyme production varies and might become significant. In many environmental assessments, the use and production of enzymes is not considered because it is only an incidental part of the overall assessment [73]. However, it has already been shown by a very simple estimation of *E-*factors that the production of enzymes can have a remarkable contribution to an overall production process [74,75]. These calculations result in *E-*factors in the order of thousands to tens of thousands of kg waste (Table 3). It should be noted that these estimates were made for small-scale production of 285 mg to 10 g of purified enzyme. It is expected that at larger scales, scaling effects and optimization of expression could decrease the *E-*factor [75]. Nevertheless, there is still limited evidence on how the production of enzymes and especially in enzyme cascades, when a higher number of enzymes is applied, affects the environmental impact of biotransformations. One of the reasons is certainly that it is a case-by-case assessment, depending on whether production is intracellular or extracellular, with optimized production organisms or natural hosts or the used chemicals for enzyme production are optimized with regard to sustainability aspects [76,77]. Nevertheless, it is clear that as the number of enzymes increases, the total mass of used biocatalyst increases, and so does the *E-*factor for their manufacturing process.

Incidentally, the same challenges can be observed in the cost analysis of enzyme cascades. The development of process-stable, recyclable enzymes significantly reduce enzyme costs per kg of product and the environmental footprint, promoting competitiveness and sustainability [78]. For high-priced pharmaceutical products, it is mainly the speed of process development and compliance with stringent regulatory requirements in the development of new synthesis routes that count. For lower-priced products, on the other hand, production costs are decisive for the final decision [79]. Here, arguments are often made about the exploitation of low-cost raw material sources for the use of enzyme cascades. In general, enzyme production can be expected to cost about 10–40 USD kg^−1^_enzyme_, depending on the expression system and enzyme [80]. Significantly higher costs of several hundred dollars can also be incurred, as estimates for a fungal enzyme have shown [81]. As a consequence, the product price is also determined by the amount of enzyme used. In the case of the synthesis of amorpha-4,11-diene, a biocatalyst productivity of 1.3 kg_product_ kg_enzyme_^−1^ was achieved for the six-step cascade with a total enzyme concentration of 0.3 g L^−1^ (calculated from [45]). Nevertheless, catalyst loadings of even more than 100 kg_product_ kg_enzyme_^−1^ are generally feasible if the process is highly optimized, which can be achieved through protein engineering and immobilization [69]. In addition, the requirements for cofactors such as NADH or ATP also increases the costs enormously (e.g., 260 USD g^−1^_NADH_, [82]). It is therefore reasonable to include regeneration enzymes in such cascades when a higher complexity is acceptable since some regeneration systems require multiple steps [83]. Further efforts will certainly focus on cofactor- and redox-neutral enzymatic cascades, which will improve the economic and environmental metrics.

## 5. Conclusions

The structural complexity of new drug compounds requires innovative and excellent organic chemistry. At the same time, the desire to develop environmentally friendly and efficient synthesis processes is growing. Biocatalysis and, in particular, enzyme cascades for multi-step, more complex transformations represent such an innovation, which at the same time represents sustainable solutions. Enzyme cascades support the achievement of green technology goals as the here-discussed examples demonstrate. The latest research in this field, such as syntheses in unconventional media, retrosynthetic approaches, or cascade modelling for better understanding and optimization, is allowing great progress in the application of enzyme cascades for the synthesis of new drugs. However, although many enzyme cascades are described catalyzing a wide variety of syntheses, performance improvements are often still needed, as well as the transfer to an industrially relevant scale. These challenges will certainly be important research tasks to be tackled in the near future.

## Figures and Tables

**Table 2:** *E-*factors and performance parameters of selected multi-enzyme cascades.

Product	Reaction Steps	*E-*Factor [kg kg^−1^]	Yield [%]	STY [g L^−1^ d^−1^]	Titer [g L^−1^]	Comments	Ref.
4-Methoxyphenyl-(1*R*,2*R*)-propanediol ^1^	2	13.8	71.2	165.0	60.1	Sequential mode	[71]
4-Methoxyphenyl-(1*R*,2*S*)-propanediol ^1^	2	21.4	37.2	41.4	33.9	Sequential mode	[71]
4-Methoxyphenyl-(1*S*,2*R*)-propanediol ^1^	2	24.3	41.7	84.4	37.4	Sequential mode	[71]
4-Methoxyphenyl-(1*S*,2*S*)-propanediol ^1^	2	45.6	19.2	18.1	17.5	Sequential mode	[71]
(1*R*,2*R*)-1-Phenyl-propane-1,2-diol ^2^	2	21.3	72 ^3^	327	55.2	Simultaneous mode	[72]
(1*R*,2*R*)-1-Phenyl-propane-1,2-diol ^2^	2	1927	58	175	43.7	Simultaneous mode, scale-up, purification	[72]
Arabinosyladenine (araA)	3	423 (1356) ^4^	53	-	3.5	Simultaneous mode	[64]

^1^ Potential anti-inflammation drug; ^2^ important building block for pharmaceuticals; ^3^ calculated from [72]; ^4^ *E-*factor of the chemical synthesis in brackets.

**Table 3:** *E-*factors for enzyme production.

Enzyme	*E*-Factor [kg kg^−1^]	Produced Amount [mg]	Comments	Ref.
crude r*Aae*UPO ^1^	4300	778	secreted from *Pichia pastoris*	[75]
purified r*Aae*UPO	18,500	295	secreted from *Pichia pastoris*	[75]
crude *Ao*FOx ^2^	2800	not specified	expressed in *Escherichia coli*	[75]
purified *Ao*FOx	4300	285	expressed in *Escherichia coli*	[75]
purified cGAS ^3^	938	10,000	expressed in *Escherichia coli*	[74]

^1^ Recombinant unspecific peroxygenase from *Agrocybe aegerita*; ^2^ Formate oxidase from *Aspergillus oryzae*; ^3^ cyclic GMP-AMP synthase from *Homo sapiens*.

## Data Availability

Not applicable.
